# Identifying and Overcoming Threats to Reproducibility, Replicability, Robustness, and Generalizability in Microbiome Research

**DOI:** 10.1128/mBio.00525-18

**Published:** 2018-06-05

**Authors:** Patrick D. Schloss

**Affiliations:** a Department of Microbiology and Immunology, University of Michigan, Ann Arbor, Michigan, USA; University of Maryland School of Medicine

**Keywords:** American Academy of Microbiology, microbiome, reproducibility, research ethics, scientific method

## Abstract

The “reproducibility crisis” in science affects microbiology as much as any other area of inquiry, and microbiologists have long struggled to make their research reproducible. We need to respect that ensuring that our methods and results are sufficiently transparent is difficult. This difficulty is compounded in interdisciplinary fields such as microbiome research. There are many reasons why a researcher is unable to reproduce a previous result, and even if a result is reproducible, it may not be correct. Furthermore, failures to reproduce previous results have much to teach us about the scientific process and microbial life itself. This Perspective delineates a framework for identifying and overcoming threats to reproducibility, replicability, robustness, and generalizability of microbiome research. Instead of seeing signs of a crisis in others’ work, we need to appreciate the technical and social difficulties that limit reproducibility in the work of others as well as our own.

## PERSPECTIVE

On first blush, one might argue that any scientist should be able to reproduce another scientist’s research with no friction. Yet, two anecdotes suffice to describe why this is not the case. The first goes to the roots of microbiology, when Antonie van Leeuwenhoek submitted a letter to the Royal Society in 1677, “Concerning little animals” (1). This seminal work and several of his prior investigations described novel observations of microorganisms, but the scientific community rejected his observations for several reasons. First, because Leeuwenhoek had little interest in sharing his methods with others, they could not be reproduced. Second, he wrote in “low Dutch,” and his writing was translated to English and edited to half its original length. This likely removed a significant amount of information regarding his methods. After several failures, Robert Hooke refined his own compound microscope and was able to reproduce Leeuwenhoek’s observations. The precision of Hooke’s observations was hindered by his use of a compound microscope, which had inferior optics to that of Leeuwenhoek’s single-lens microscope. In the process, Hooke popularized the compound microscope. This succession of events is illustrative of many of the current problems that microbiologists face in validating each other’s work. Time has proven that Leeuwenhoek’s work was rigorous, impactful, and robust. It was not sloppy, and there was no fraud. But, it required multiple efforts by one of the greatest minds in science to reproduce the results, and even then it was a poor reproduction of the original.

The second anecdote took place more recently. In 2011, Philip Bourne challenged those attending the “Beyond the PDF” workshop (https://sites.google.com/site/beyondthepdf/) to reproduce the analysis performed in his group’s 2010 study “The Mycobacterium tuberculosis drugome and its polypharmacological implications” (2). The response to that challenge resulted in a collaborative analysis involving the original authors and scientists from Spain, China, and the United States that challenged concepts critical to understanding reproducible research (3). The reanalysis demonstrated that the value of reproducibility, the degree to which research should be reproducible, the amount of effort required to reproduce the research, and who should be able to reproduce the research are questions without simple answers. Bourne’s track record in science and as a leader in the field of bioinformatics suggests that his group was not sloppy, and his challenge indicated a level of transparency that is rare in science. Yet, the investigators who sought to reproduce the findings found that someone with basic bioinformatics skills would require at least 160 h to decipher the approaches used in the original analysis and an additional 120 h to implement them to complete the reproduction.

Both of these anecdotes are at odds with the tone of a recent report by the American Academy of Microbiology’s (AAM’s) 2015 colloquium “Promoting Responsible Scientific Research” and its accompanying editorial in *mBio* (4, 5). The report is a useful lens into how microbiologists view the reliability of research in their field. The colloquium identified “(i) sloppy science, (ii) selection and experimental bias, and (iii) misconduct” as the primary contributors to the ongoing problems with ensuring the reliability of microbiology research. Although the participants were quick to point out that misconduct was a relatively minor contributor to the problem, the four case studies that accompanied the original report all concern misconduct. Missing from these reports was any of the nuance or humility enveloped in Leeuwenhoek’s case or Bourne’s challenge: ensuring that one’s research design and methods are sufficiently clear is enormously difficult. Researchers are frequently frustrated with their own lack of documentation when they are contacted about a forgotten detail years after a paper is published. Put simply, most problems with reproducibility are not due to sloppy science, bias, or misconduct. I contend that many of the difficulties that we face in ensuring the reproducibility of our research are social and driven by cultural forces within science.

Although the issues identified by the AAM colloquium participants are important, this Perspective argues that they are not the main reason for a reproducibility crisis in microbiology. It is scientifically valuable to consider what other factors threaten our ability to reproduce a result. Although these factors highlight the technical limitations and cultural forces that we face, our inability to validate a result may also indicate that we still have much to learn about biology. Furthermore, we must remember that whether we can validate a result is not just a product of rigorous scientific practice but also a product of stochastic forces (6, 7). We must also be on guard against assuming that just because a result is reproducible that it is correct (8). With these general points in mind, the goals of this Perspective are threefold. First, I present a framework for thinking about how science is conducted within the microbial sciences. Second, I provide an overview of various factors that threaten the field’s ability to validate prior results and the tools that we can use to overcome these problems. Third, based on these issues, I provide five exercises that research groups can use to motivate important discussions of their practices and how their practices foster or impede efforts to validate the researchers’ results. Although I will primarily focus on examples from microbiome research, the principles are generalizable to other areas of microbiology, as all scientists struggle to ensure the reproducibility of their research.

## THREATS TO REPRODUCIBILITY

### Developing a framework.

One of the struggles in discussing reproducibility, replicability, and the factors that can limit them is agreeing upon how they should be defined (7). Reproducibility is used as a vague term for being able to repeat another researcher’s work whether that is with the same protocols or with the same populations. This Perspective will use definitions that have greater precision and that are based on definitions that are widely used in the statistics literature. Reproducibility is the ability to regenerate a result with the same data set and data analysis workflow, and replicability is the ability to produce a consistent result with an independent experiment asking the same scientific question (8). I propose a similar framework that accounts for the practice of applying multiple methods to the same samples to improve the robustness and generalizability of a result (Table 1) (9). It is critical for scientists to give attention to the right-hand column of the framework. Most research is exploratory, and scientists, editors, and funding agencies generally lack the will or ability to confirm previous studies via independent replications or attempts to generalize results in other model systems or human populations (4, 5, 7, 10, 71). Results must be reproducible and robust, but they also need to be replicable and generalizable.

**TABLE 1:** Simple grid-based system for defining concepts that can be used to describe the validity of a result^a^

Methods	Same experimental system	Different experimental system
Same methods	Reproducibility	Replicability
Different methods	Robustness	Generalizability

a This is a generalization of the approach used by Whitaker (9), who used it to describe computational analyses.

### An example.

The question of whether there are microbiome-based signatures of obesity is a useful illustration to demonstrate the factors that affect each of the quadrants of the grid in Table 1, and it can be used to underscore the difficulty of ensuring the reproducibility, replicability, robustness, and generalizability of results. Several research groups, including mine (11), have attempted to validate the result that obese individuals were more likely to have lower bacterial diversity and relative abundances of *Bacteroidetes* (12, 13). The original observation was published in 2008 using 16S rRNA gene sequence data and continues to engender much enthusiasm for the role of the microbiome in human health (14). It is important to note that the original study was one of the first to use high-throughput amplicon sequencing, and so there was minimal infrastructure to deposit and store such sequences in public databases. Furthermore, many of the software tools that we now rely on for facilitating reproducible workflows were not available. Regardless, although the original study was performed using poorly described data curation methods, we were able to independently obtain the same results as the original study when using the same data set. The original result can thus be considered reproducible (Table 1). However, when we used the same methods with data from nine other cohorts, we and others have failed to replicate the result (11–13). These failures to replicate the original result may be due to methodological differences across the replicating studies, differences in study populations, or statistical variation. Our study demonstrated that each of 10 cohorts was significantly underpowered to identify a 10% difference in Shannon diversity (11). Therefore, the lack of statistical power may have been responsible for an inability to detect a difference. Each of these studies was rather large for the time that it was published within the development of the microbiome research field, and so the original researchers likely thought that they had obtained the best statistical power that was feasible. Identifying what is a biologically meaningful difference in any parameter within the microbiome literature to complete a meaningful power analysis has been a challenge. Each of these factors still makes it nearly impossible to perform a meaningful *a priori* power analysis to aid in the design of any cohort. Next, it is worth noting that those involved in the original study pursued multiple approaches to better understand the question of whether the microbiota is important in obesity. They initially sought microbiome-based signatures using mouse models (15). They observed stark differences in the microbiota of genetically lean and obese mice and found that the microbiota of obese mice could transmit the propensity to gain weight to germfree mice (15). In a human cohort, they generated multiple data sets that each reflected different regions of the 16S rRNA gene. In obese individuals, they observed lower diversity and relative abundance of *Bacteroidetes* (14). They also used shotgun metagenomic sequencing to postulate the enrichment of carbohydrate processing genes in obese individuals (14). In a smaller cohort study, although the subjects’ diversity remained constant, as the authors predicted, the relative abundance of *Bacteroidetes* increased as the subjects lost weight (16). Although each part of their approach had significant weaknesses, including methodological biases and underpowered experimental designs, their results supported the hypothesis that there are microbial signatures associated with obesity. This conclusion was robust within the cohort that they studied, but it was not generalizable to other cohorts. Within this example, it is apparent that scientists acted in good faith given the technological and cultural conditions that they were working under. These conditions underscore the difficulty of replicating and generalizing results.

### Reproducibility.

Threats to reproducibility are some of the most fundamental and easiest in which to lay fault on the original investigators. If a result cannot be reproduced, then it is difficult to have confidence that it can be replicated or generalized. Thus, the ability to reproduce a result is critical.

Too often, the underlying raw sequencing data and associated data that contextualize the sequencing data are not accessible. Clearly, this makes reproducing a prior analysis impossible (17, 18). Well-established databases for storing a variety of “omics” data exist, and other data should be archived in third-party databases such as figshare (https://figshare.com) and Dryad (https://datadryad.org). However, some researchers still fail to post their sequencing data to public databases or do not provide the necessary metadata with the sequencing data. As we developed the obesity meta-analysis, we were dependent on the original authors to provide the information for two of the 10 data sets. Furthermore, the data made available from the original study provided only the subjects’ body mass indexes (BMIs) as categories (14). We were unable to access the actual heights, weights, and BMIs. We did not include three large data sets from two studies because their data were inaccessible due to onerous data sharing agreements (19, 20). Two other data sets required at least a month of effort to obtain (21, 22). More broadly, Stodden et al. (23) recently showed that although *Science* magazine has had clear guidelines requiring authors to make the data and code for their studies available, only 44% of the authors who published papers in 2011 and 2012 were willing to provide the resources. Lack of access to the data and underlying code for an analysis clearly limits the ability of others to reproduce and build upon that analysis.

“Link rot”—the fact that web or e-mail addresses become deprecated—is a significant problem for those attempting to access the data and methods needed to reproduce a result (24). Changes in institutional affiliation frequently render e-mail addresses invalid. ORCID (https://orcid.org) has emerged as a technology to solve the e-mail rot problem, and many journals use it to provide a persistent link to an individual’s many scientific identities over their career. The fraction of manuscripts including web resources continues to grow, and yet at least 70% of those manuscripts include URLs that are inaccessible (24). To prevent link rot, services like Zotero (https://www.zotero.org) can provide a digital object identifier (DOI) that persists even if the link that it points to changes. Unfortunately, the developer of the web resources must ensure that the resource remains active. The inevitability of link rot further emphasizes the importance of using public and stable servers that are likely to persist.

Related to link rot, rapid advances in sequencing technology, data curation, databases, and statistical techniques present an additional threat to reproducibility because resources and what are considered best practices are constantly evolving. This evolution is not always well documented. For example, the mothur software package has had 40 major updates since it was originally released in 2009 (25). The RDP (26) (http://rdp.cme.msu.edu) and SILVA (27) (https://www.arb-silva.de) databases that many use as a reference for aligning and classifying 16S rRNA gene sequences are updated annually, and the popular Greengenes database files have not been updated since 2013 (28) (http://greengenes.lbl.gov and http://greengenes.secondgenome.com). With each release, curators expand the number of sequences in the database and make modifications to their taxonomic outline. For software and databases, it is critical that authors report version numbers if there is to be any hope of replicating previous work. Unfortunately, the reliance on web-based resources and workflows at sites such as GenBank (https://www.ncbi.nlm.nih.gov/genbank), Greengenes, RDP, and SILVA precludes analyzing new data with older versions of the sites. The Greengenes website removed their online tools in April 2017, exemplifying the problem with web-based workflows. Their database files are now available through the company Second Genome, but their tools are not. Combined with the development of new sequencing platforms and deprecation of old platforms, these changes in technology, references, and software underscore the importance of adequately documenting workflows and enabling users to recreate the conditions that the original researchers worked under.

Because many journals impose word limits on manuscripts, Materials and Methods sections become a chain of citations to previous works that each cite previous work (10). Improved documentation in supplementary materials or archives such as protocols.io (https://www.protocols.io) for lab-based methods or through GitHub (https://github.com) for data analysis workflows would make it easier for researchers to avoid these rabbit holes. For data analysis workflows, software such as GNU Make (https://www.gnu.org/software/make/) and the Common Workflow Language (29) make it possible to track data dependencies and automate a workflow. For example, we used GNU Make to write a workflow in our meta-analysis of the obesity data such that downloading a copy of the scripts from the project’s GitHub repository and writing “make write.paper” in the command line will reproduce our analysis. Although considerable effort is required to make them work, workflow tools make it possible to trace the provenance of a summary statistic from the manuscript back to the raw data.

The use of workflow tools, literate programming tools (e.g., RMarkdown [30] and Jupyter [31]), and version control software provides researchers with mechanisms to track the development of their analyses. Furthermore, these tools can help researchers reflect the fact that their analysis was not a linear process resembling a pipeline. In reality, questions change and scientists can fall into the traps of the “Garden of Many Forking Paths,” where they go looking for a desired result (32), or “*P*-hacking,” where large numbers of statistical hypothesis tests are attempted without adequately correcting for performing multiple tests (33). Although it is possible to preregister data analysis plans (34–36), these plans are often too stringent for most exploratory research. An increasing number of microbiome researchers are using workflow, literate programming, and version control tools to document their analyses. I have yet to observe widespread exploration of the history of projects’ repositories or the adoption of preregistration of data analysis plans among microbiome researchers. Although these have their technical and cultural limitations, they offer greater transparency to improved reproducibility.

### Replicability.

A number of threats similar to those for reproducibility could explain why a previous result cannot be replicated. In addition to those detailed previously, there are threats related to differences in systems or populations and the ability to control for those differences.

Forgotten in discussions of replication failures by many microbiologists is that a replication may fail because replication is statistical rather than deterministic (6). Every experiment has a margin of error, and when the effect size is near that margin of error, it is likely that a statistically significant result in one replicate will not be significant in another. Most researchers use a frequentist null model hypothesis testing approach with which they are willing to accept a type I error of 0.05. Stated more colloquially, they are willing to incorrectly reject a null hypothesis in 5% of the replicates. Further, they rarely quantify the risk of falsely accepting a null hypothesis (i.e., type II errors) (37). In some cases, an insufficient sample size in the replicate study may explain the failure to replicate a study. In other cases, the original study may have been underpowered, rendering it susceptible to an inflated risk of type I errors (38). Solutions to these problems include preregistering data analysis plans (34–36), justifying sample sizes based on power calculations (10, 11, 37), and using Bayesian frameworks that allow prior knowledge of the system to influence the interpretation of new results (39, 40). It needs to be underscored, however, that to measure statistical power and use that information to inform sample size selections, one must know what a biologically relevant difference is. The microbiome field has yet to make that determination. Our previous power analysis used various differences in Shannon diversity (11). As we indicated, those levels were picked because they seemed reasonable, not because of a biological foundation. Furthermore, there was no reason to think that diversity metrics are the most biologically meaningful parameters to base the calculations on.

Beyond problems of sample size and statistical power calculations, problems with experimental design are also often a threat to replicability because investigators fail to account for confounding variables in the original study. A subsequent study may fail to find the same result because its design is not impacted by the confounding variable. In sequence-based analyses, threats to replicability are encountered when samples are not randomized across sequencing runs. These so-called batch effects have been a problem with a large number of analytic techniques beyond sequencing (41). One notable example occurred within the Human Microbiome Project where 150 people were recruited in Houston, TX, and 150 were recruited in St. Louis, MO (21). Researchers at the Baylor College of Medicine and Washington University performed the DNA extractions for the two sets of subjects, respectively. Researchers at the Baylor College of Medicine, the J. Craig Venter Institute, and the Broad Institute sequenced the DNA from the Houston subjects, and researchers from Washington University sequenced the DNA from the St. Louis subjects. The subject’s city was the variable with the largest effect size, although all parties used the same standard operating procedures to sample the subjects and extract and sequence the DNA (21, 42). Because the city of origin and the center that did the extractions were perfectly confounded, it was impossible to quantify the impact of geographic differences on the microbiome. Instead of being a single study that intended to address associations between geographic and microbiome variation, this became two replicate studies that were unable to address the influence that geography has on the microbiome. It is easy to blame those who designed the study for this confounding, but it is important to acknowledge the social conditions that were resolved via negotiations that may have impacted the design and the need to garner buy-in from different centers.

In addition to variation between human cohorts, variation between bacterial and model organism strains can hinder efforts to replicate results. In microbiome research, it is widely appreciated that the microbiota of research animals from the same litter and breeding facility are largely clonal and distinct from those in other facilities (43, 44). Mice from two breeding facilities at the same institution may have completely different microbiota. The best example of this phenomenon is the presence of segmented filamentous bacteria in mice purchased from Taconic Farms but not Jackson Laboratories (45, 46). Thus, the origin of the mice and not the experimental treatment may explain the roles ascribed to the microbiota. This is particularly a problem for genetic models when researchers obtain mutant animals and animals with the wild-type background as their control. In such cases, using the offspring of heterozygous matings is critical (47). Similarly, comparing the microbiota of obese and lean individuals from a cohort of twins and their mothers in Missouri (14) may have confounding factors that differ from members of Amish communities (22). In these cases, the problem with replicability is due not to the quality of the investigator’s experimental practices but to the differences that may be biological, demographic, or anthropological. Thus, failure to replicate a study across different strains or cohorts could suggest that other interesting factors play a role in the phenomenon under study.

Just as uncertainty over the variation in mouse and human populations can impact the replicability of results, uncertain provenance and purity of reagents, organisms, and samples can also threaten replicability. Perhaps the best-known example is the discovery that HeLa cells contaminate many other cell lines, especially those in the same laboratory (48, 49). Similarly, investigators frequently realize that they are working with bacterial strains that were incorrectly typed or that have evolved during serial passages from the freezer stock (50, 51). Short of resequencing the cells, experimental controls, limiting the number of passages from freezer stocks, and periodic phenotyping of the strains can help to overcome these problems. However, it is part of our scientific culture that if a colleague sends a strain to another researcher, the recipient generally trusts that they get the correct strain. There is also a growing awareness that DNA extraction kits can be contaminated with low levels of bacterial DNA (52). These contaminants have led to the identification of contaminants as being important members of the lung and placental microbiota when mock extractions are not sequenced in parallel (53–55). For each of these threats to replication, we would be well served by following the proverb to “trust, but verify” by testing the robustness of the results.

### Robustness.

Every method has its own strengths and weaknesses. Therefore, it is important to address a research question from multiple and hopefully orthogonal directions. This strategy combines the strengths of different methods to overcome their individual weaknesses (56). Evaluating the robustness of a result from a single cohort is becoming more common as researchers pursue multiple approaches, including 16S rRNA gene sequencing, metagenomics, metatranscriptomics, and metabolomics (57–59). Of course, biases in the underlying cohort design, sample collection and storage, or the nucleic acid processing will propagate through the analyses. The way to remedy this is to select methods that are as independent from each other as possible. For example, data collected from multiple regions of the 16S rRNA gene would not be considered truly independent data sets since amplicon sequencing would have been applied to the same samples. The results would be marginally more independent if one were to layer shotgun metagenomic data onto the 16S rRNA gene sequence data, because although the same DNA would be used for sequencing, metagenomics provides information about the genetic diversity and functional potential of a community rather than the taxonomic diversity of a community. Metabolomic data would be even more independent from the DNA-based methods since they require completely different sample processing steps. Quantitative PCR, cultivation, and microscopy could be similarly layered on these data. Ultimately, it is impossible for the results of each set of methods to be fully independent. If the underlying design of the study is flawed by insufficient statistical power or failure to account for confounding variables, then any attempts to test the robustness of a result will also be flawed.

### Generalizability.

A motivating goal in science is to have a result that is generalizable across populations or systems. Within a scientific culture that does not place value on publishing negative results, it is difficult to assess whether scientists’ bias to support their prior results affects the ability to claim that a result is robust or generalizable. Similarly, failing to attempt replication studies hinders the ability of researchers to test the generalizability of most results. Scientists often fear being “scooped” (60). In reality, it is the second researcher who examines the same question who has the opportunity to increase the field’s confidence that a result is valid (61). Generalizability is an important and broad question. Model organisms (e.g., Escherichia coli) and strains of those organisms (e.g., K-12) have taught us a great deal about the biology of those organisms. However, it is not always trivial to generalize that knowledge to related species and strains or from *in vitro* to *in vivo* conditions and on to human subjects (62, 63). Like a failure to reproduce, replicate, or demonstrate the robustness of a result, a failure to generalize a result is not a failure of science. Rather, it is an opportunity to better understand the complex biology of bacteria and how they interact with their environments.

## FOSTERING A CULTURE OF GREATER REPRODUCIBILITY AND REPLICABILITY

### Training.

Throughout my discussion of the threats to reproducibility, replicability, robustness, and generalizability, failures on the part of scientists to be more transparent, provide greater documentation, or design better experiments have been balanced by an appreciation that we work within a scientific culture. This culture is limited by our ignorance of biology, rapid expansion in technology, misaligned rewards, and a lack of necessary training. A key observation from the work of Garijo and colleagues (3) was that the level of detail needed to reproduce an analysis varies depending on the researcher’s level of training. An expert in the field understands the nuances and standards of the field, whereas a novice may not know how to install the software. This highlights the need for training. Yet, many microbiology training programs focus on laboratory skills while ignoring data analysis skills. A number of excellent “best practices” documents have emerged in recent years (64–69). In addition, organizations, including Software Carpentry and Data Carpentry, offer workshops to introduce researchers to the best practices in reproducible research (70) (https://carpentries.org). Massively open online courses have been developed that teach scientists best practices for performing reproducible analyses. The most popular of these is a training program from faculty at the Johns Hopkins Data Science Lab (http://jhudatascience.org). Just as important as learning the fundamentals of how to implement reproducible research methods is honing those skills in one’s research. A novice could not reproduce Beethoven’s “Für Elise” from sheet music without prior experience playing the piano. Similarly, novices cannot expect to reproduce a result without learning the methods of their discipline. With this analogy in mind, I have created the *Riffomonas* project, which expounds on the threats to reproducibility and tools that microbiome researchers can use to maximize the computational reproducibility of their analyses (http://www.riffomonas.org). The *Riffomonas* materials use microbiome-related examples to illustrate the importance of transparency, documentation, automated workflows, version control, and literate programming to improving the computational reproducibility of an analysis. The goal is that once scientists have been trained in these practices, they can apply them to their own work and use them to “riff” or adapt and build on the work of others.

### Exercises.

The following exercises are meant to motivate conversations within a research group to foster a culture improving reproducibility and replicability and to underscore the threats outlined above.

1. Working away from each other, have two or more people write instructions on how to fold a paper airplane. Have the participants trade instructions, separate, and implement the instructions. After the participants come back together, ask the following questions. How closely did the final airplanes resemble that of the person who developed the instructions? What would have helped to make the reproductions more faithful? How much did the author of the instructions assume about the other person’s prior knowledge of paper airplanes, resources, and abilities? What challenges would length limitations place on this exercise? How does this exercise resemble the descriptions in the Materials and Methods section of papers for standard methods (e.g., PCR) and for novel methods (e.g., bioinformatic workflows)?
2. Imagine that a graduate student is really excited about an analysis that you performed in your most recent paper and would like to replicate the analysis with their own data. But first, they want to make sure that they reproduce your results. What steps are likely to cause the student problems? If it is not clear to you what problems they might face, find your favorite figure from a paper by a different research group than your own. Can you reproduce the figure? What is standing in your way?
3. Take a figure from your recent paper and improve the likelihood that another researcher would be able to reproduce it. Where are the data, and how would the researcher access them? What calculations were performed to summarize the data? What software was used to generate the figure? Is that software freely available? What steps would the researcher need to take to generate the figure? When you write your methods, what experience level are you writing for? Whom should you be writing for? When you are confident that you have made the figure as reproducible as you can, give the instructions to several colleagues and ask for their feedback.
4. Complete an audit of the reproducibility practices in your research group. Table 2 provides a rubric that someone working within the host-associated microbiome field might use to assess their research. Within your research group, modify this rubric to suit your needs. For your next paper, work to improve one element from the rubric and constantly be developing an ethic of fostering greater reproducibility.
5. Many of the threats to reproducibility and replicability are a product of scientific culture: methods sections are terse or vague, original data are not available, analyses rely on expensive and proprietary software, analysis scripts are available “upon request from the authors,” and papers are published behind paywalls. Some might give into despair thinking that one person or research group can have only a minor impact. Have a discussion within your group about why things are this way, whether your group’s practices should change, and what would be the easiest and most impactful thing to change.

**TABLE 2:** An aspirational rubric for evaluating the practices that host-associated microbiome researchers might use to increase the reproducibility and replicability of their work^a^

Practice	Good	Better	Best
Handling of confounding variables	Prior to generating data, did we identify a list of possible confounding variables—biological and technical—that may obscure the interpretation of our results?	Do we indicate the level of randomization and experimental blocking that we performed to minimize the effect of the confounding variables?	Does the interpretation of our results limit itself to only those variables that are not obviously confounded?

Sex/gender as confounding variables	Do we indicate the sex/gender of research animals/participants?	Do we provide a justification for the lack of even representation?	Is there equitable representation of sexes/genders? Do we account for them as a variable?

Experimental design considerations	Do we have an active collaboration with a statistician who helps with experimental design and analysis?	Do we indicate the number of hypothesis tests that we performed and have we corrected any *P* values for multiple comparisons?	For our primary research questions, have we run a power analysis to determine the necessary sample size?

Data analysis plan	Before starting an analysis, have we articulated a set of primary and secondary research questions?	Has someone else reviewed our data analysis plan prior to analyzing the data?	Have we registered our data analysis plan with a third party before starting the project?

Provenance of reagents	Is there a table of reagents such as cell lines, strains, and primer sequences that were used?	Where possible, have we obtained reagents from certified entities like the American Type Culture Collection (ATCC)?	Is there a statement indicating how we know the provenance and purity of each cell line and strain?

Controlling for initial microbiota	Are mice obtained from a breeding facility that allows us to track their pedigree?	Where possible, are mice from different treatment groups cohoused to control for differences in initial microbiota?	Are comparisons between mice with different genotypes made using mice that are the result of matings between animals that are heterozygous for that genotype?

Clarity of software descriptions	Are all methods, databases, and software tools cited? Do we follow the relevant licensing requirements of each tool?	Do we indicate dates and version numbers of websites that were used to obtain data, code, and other third-party resources?	Are detailed methods registered on a website like protocols.io or GitHub?

DNA contamination	Did we quantify the background DNA concentration in our reagents? Did we sequence an extraction control?	Are we taking steps to minimize reagent contamination?	What methods do we take to confirm a result that a sequencing result may be clouded by contaminating DNA?

Availability of data products	Are all of the raw data publicly available?	Are intermediate and final data files publicly available?	Are tools like Amazon Machine Images (AMIs) used to make a snapshot of our working directory?

Availability of metadata	Are all of the metadata necessary to repeat any analyses that we performed publicly available?	Have we adhered to standards in releasing the minimum amount of metadata about our samples?	Did we go beyond the minimum to incorporate other pieces of metadata that will inform future studies?

Data analysis organization	Are all data, code, results, and documentation housed within a monophyletic folder structure on our computer?	Is this project contained within a single directory on our computer, and does it separate our raw and processed data, code, documentation, and results?	Is this folder structure under version control? Is the project’s repository publicly available? Are there assurances that this repository will remain accessible?

Availability of data analysis tools	Are free and open tools used in preference to proprietary commercial tools?	Is the computer code required to run analyses available through a service like GitHub?	Are Amazon Machine Images or Docker containers used to allow recreation of our work environment?

Documentation of data analysis workflow	Is our code well documented? Do we use a self-commenting coding practice?	Does each of our scripts have a header indicating the inputs, outputs, and dependencies? Is it documented how files relate to each other?	Are automated workflow tools like GNU Make and Common Workflow Language used to convert raw data into final tables, figures, and summary statistics?

Use of random number generator	Do we know whether any of the steps in our data analysis workflow depend on the use of a random number generator?	For analyses that utilize a random number generator, have we noted the underlying random seed?	Have we repeated our analysis with multiple seeds to show that the results are insensitive to the choice of the seed?

Defensive data analysis	Is our data analysis pipeline flexible enough to add new data?	Does our code include tests to confirm that it does what we think it does?	Did we make use of automated tests and continuous integration tools to ensure internal reproducibility?

Ensuring short- and long-term reproducibility	Did we release the underlying code and new data at the time of submitting a paper with their DOIs and accession numbers?	Did we include a reproducibility statement or declaration at the end of the manuscript? Are ORCID identifiers provided for all authors?	What mechanisms are in place to ensure that our analysis remains accessible and reproducible in 5 years?

Open science to foster reproducibility	Have we released any embargoes on our code repository and raw data prior to submitting the manuscript?	Did we post a preprint version of our manuscript prior to submission?	Have we published under a Creative Commons license? Is a permissive reuse license posted with our code?

Transparency of data analysis	Is it clear where one would go to find the data and processing steps behind any of our figures?	Are electronic notebooks publicly accessible, and do they accompany the manuscript?	Were literate programming tools used to generate summary statistics, tables, and figures?

^a^ Although many of the questions can be thought of as having a yes-or-no answer, a better approach would be to see the questions as being open ended with the real question being “What can we do to improve the status of our project on this point?” With this in mind, a researcher is unlikely to have a project that satisfies the “Best” column for each line of the table. Researchers are encouraged to adapt and modify the categories to suit their own needs.

## CONCLUSION

A motivating concept that has been attributed to many people to improve the reproducibility of one’s research is that they should think of themselves from a month ago as their most important collaborator. They are not available to answer questions to things that they have forgotten in the intervening period. This is a common occurrence for many researchers who put projects to the side for a time to prepare for examinations, go on vacations, or work on other projects. Trying to piece together what they did previously is often a frustrating process. If instead they had been using tools to improve reproducibility, then they will be doing themselves a favor when they return to the project. Similarly, I consider their supervisor or coauthors to be their second most important collaborators. It is likely that the corresponding author was not the person who implemented the details of the analysis plan. Thus, it is important that they have access and the ability to navigate the project when they receive a query about how the analysis was done. Anyone who has done research can attest to how difficult it can be to satisfy these two sets of “collaborators.” And yet, if we can satisfy these collaborators, then we should be able to satisfy the third collaborator, the reader who hopes to build upon our work to generalize it or go in a new direction.

It is important to see that attempts to guard against threats to reproducibility, replicability, robustness, and generalizability are positive forces that will improve science. They have been considered a form of scientific “preventative medicine” (8). Although guarding against these threats is not a guarantee that the correct conclusion will be reached, the likelihood that the result is correct will be increased. Beyond ensuring “correctness,” the goal of these efforts, and I would argue their primary goal, should be to enable future scientists to build upon the work to go further. Before attributing difficulties with reproducibility, replicability, robustness, and generalizability to a dim view of our fellow scientists as being sloppy, biased, or untrustworthy, it is worth seriously considering the many factors—biological, statistical, and sociological—that pose a threat. Although there is much room for improvement, we must acknowledge that science is a process of learning and that it is really freaking hard.
